# Our Journal

**Published:** 1868

**Authors:** 


### EDITORIAL AND MISCELLANEOUS.

### OUR JOURNAL.

This is the first Number of Vol. IX, new series. We hope it will suit the views of our patrons, and that they will continue to smile upon us in the future as in the past, and give us the hearty approval we are trying so earnestly to deserve. By this arrangement we reduce our expenses 25 per cent., and our patrons have a like reduction in the price of the Journal, whilst they get nearly the same amount of reading matter in the bi-monthly they formerly got in the monthly. In other words, one number of the bi-monthly contains nearly as much reading matter as two numbers of the monthly.

We beg to say to our patrons that we are far from being discouraged. We believe there is a better day coming, and for that better day we will work, and hope, and wait.

We are not young, nor yet have we faded into the *sear and yellow leaf;* but no matter how this may be, we believe we shall live to see the end of burdensome taxation, military rule, stamp taxes, internal revenue officers, and the swarm of free-booters, plunderers and spies that infest the country from the Potomac to Texas. We believe we shall yet see the glorious sun shed down his prolific radiance upon the golden harvests, and grassy plains, and waving forests of a prosperous and happy people, whose patient endurance of wrong shall have extorted her deliverance even from her enemies. We expect to see all this and much more. The husbandman will sow and reap where desolation now reigns. The vine-dresser will erect his trellis and gather from the fruitful scuppernong, unequaled by oriental fable, a vintage fit for kings. Churches, school houses and colleges, swept to desolation by the wrathful fury of passion and war, will rise like the Phœnix from her ashes, and religion and science, twin sisters of human happiness and hope, shed their benign and irradiating influence all over the land. Then let us hold up our heads, nor abate one jot or tittle of our diligence in calling back the dignity, beauty and glory of our beloved land. Let us plant again our fruit trees and vineyards. Let gentle hands train the multiflora and cypress, and inhale the perfume of the violet and rose, before the dew, yet fresh from mystic springs, gives its sweetness to the zephyr and the sun.

We say again, let us faint not, but gathering new strength and a loftier courage, rise to the dignity of the crisis, bearing a manly part in upholding our Christian civilization, and in fulfilling a mission sublime in the objects it contemplates, being nothing less than the preservation of science, literature, religion and social progress from the inert despair which now broods like a pall over the fairest portion of the universe.

It is now boldly asserted that Southern civilization was a myth; that they have contributed little to literature, to science, or the arts; that they lack thoroughness in everything; and that those who do aspire to great ends must seek mental food elsewhere than at home. Shall we give countenance to this monstrous libel by permitting our own institutions to languish, nay, to perish, under the plea of hard times? We know you are poor; we also are poor. Upon your devoted heads fell the fury of a four years war; and so it did upon ours. With you we marched through snows and rains without tents, and almost without food or clothes, to find ourselves destitute, at the close of the war, of even a pocket-knife; and yet we have lived. Many times within a year we have not had a half day's supply of food for a family of eight persons; and yet we have never failed to publish the Journal.

Let our friends strengthen our hands as they may be able, and we will put to the blush the monstrous assumptions of our enemies.

